# Laser Fabrication of Nanoholes on Silica through Surface Window Assisted Nano-Drilling (SWAN)

**DOI:** 10.3390/nano11123340

**Published:** 2021-12-09

**Authors:** Yu Lu, Lin Kai, Qing Yang, Guangqing Du, Xun Hou, Feng Chen

**Affiliations:** 1 State Key Laboratory for Manufacturing System Engineering and Shaanxi Key Laboratory of Photonics Technology for Information, School of Electronic Science and Engineering, Xi’an Jiaotong University, Xi’an 710049, China; zjkly19900714@126.com (Y.L.); 3120105005@stu.xjtu.edu.cn (L.K.); guangqingdu@mail.xjtu.edu.cn (G.D.); houxun@mail.xjtu.edu.cn (X.H.); 2 School of Mechanical Engineering, Xi’an Jiaotong University, Xi’an 710049, China; yangqing@mail.xjtu.edu.cn

**Keywords:** femtosecond nano-fabrication, Bessel beam, surface window assisted nano-drilling

## Abstract

Nano-structures have significant applications in many fields such as chip fabrications, nanorobotics, and solar cells. However, realizing nanoscale structures on hard and brittle materials is still challenging. In this paper, when processing the silica surface with a tightly focused Bessel beam, the smallest nanohole with ~20 nm diameter has been realized by precisely controlling the interior and superficial interaction of the silica material. An effective surface window assisted nano-drilling (SWAN) mechanism is proposed to explain the generation of such a deep subwavelength structure, which is supported by the simulation results of energy depositions.

## 1. Introduction

Reducing the structure feature size in nano-fabrication plays an essential role in many important areas. For example, nanoscale robots can deeply access complex and narrow regions inside the human body, which holds great significance in many biomedical applications such as drug delivery, disease diagnosis, and microsurgery [1,2,3]. What is more, reducing the feature size of a single device can improve its density, which will improve the calculating speed and the storage density of electronic chips [4,5]. Among the varying nano-fabrication methods, femtosecond laser direct writing has been a powerful tool in recent years due to its high accuracy, high flexibility, and ability in processing nearly any kind of material [6,7,8,9]. In femtosecond laser processing, the feature size of the nanostructures acquired is confined by the diffraction limits, which means that the laser spot must be comparable to the laser wavelength. To overcome this fundamental limit, much excellent work has been performed. In recently reported work, nanostructures with ~10 nm feature sizes can be realized on photosensitive polymers [10,11], semiconductors [12], or some dielectric films [13]. On the other, hard and brittle materials such as ceramics, sapphire, and glass are widely applied in harsh and extreme environments such as outer space. However, hard and brittle materials often hold high thermal and chemical stability, wear resistance, and low fracture toughness [14,15,16], which make this kind of material difficult to fabricate. As a result, obtaining nanostructures on the surface of these materials is still challenging [17,18].

In this paper, on silica, a classic hard and brittle material, nanohole structures with 20 nm feature size have been realized by a single focused femtosecond pulse shaped by an axicon, through which a Gaussian beam can be shaped into a Bessel beam. Previous research on laser fabrication with a Bessel beam have often focused on acquiring high long-width ratio channels with sub-μm diameter inside the materials [19,20,21,22,23,24]. In this work, the Bessel beams are often buried deeply under the boundaries. During the laser–material interaction processes, the area excited by the focused Bessel beam is surrounded by cold and dense materials. In this condition, the damage mechanisms are a micro-explosion caused by very high energy concentrations [20,25]. Here, by moving a tightly focused Bessel beam to the silica surface, the boundary effects will play a key role during the laser–material interaction. The newly induced mechanisms, which are concluded as a surface window assisted nano-drilling (SWAN) effect, are analyzed by the numerical study of the energy deposition process. The newly proposed SWAN mechanism can lead to a series of nanostructures in which nanohole structures with the smallest diameter of 22.7 nm can be observed.

## 2. Materials and Methods

**Figure 1:**
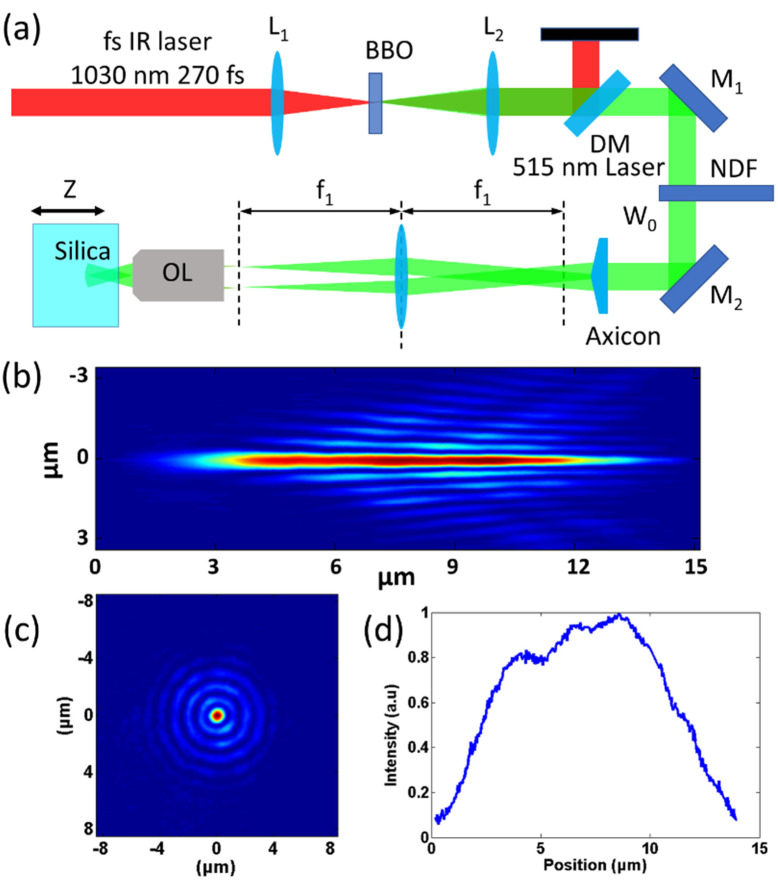
(**a**) schematic diagram of the Bessel beam assisting the nanofabrication system. The BBO generates a 515 nm laser. A dichroic mirror separates the 1030 and 515 nm lasers. An axicon shapes the 515 nm laser beam into the Bessel beam. Long-focal lens and objective project compress the Bessel beam onto the silica sample. L: lens; DM: dichroic mirror; M: mirror; NDF: neutral density filter; OL: objective lens. (**b**) Longitude and (**c**) radial intensity distribution of the Bessel beam. (**d**) The energy distribution of the center spot Bessel beam along the propagation direction.

The schematic diagram of the fabrication system is shown in Figure 1a. A femtosecond fiber laser (FemtoYL-20, YSL Photonics Co. Ltd, Wuhan, China, 1030 nm center wavelength, 270 fs pulse width) generates fs pulses. A lens L_1_ focuses the IR femtosecond beam onto the BBO crystal to acquire a 515 nm laser, which is collimated by lens L_2_. A dichroic mirror separates the 515 and 1030 nm laser and the 1030 nm laser is then blocked. Incident pulse energy can be adjusted by a neutral density filter. The 515 nm laser with a diameter of ~2 mm passes an axicon (AX251-A, Thorlabs Inc, Newton, MA, United States, 1.0° physical angle) to shape the incident light into the first Bessel beam with a half conical angle of 0.45°. The L_3_ (160 mm for effective focal length) and the objective lens (×100, 0.8 NA) compose a 4f system. The Bessel beam is projected and compressed onto the silica sample through the lens L_3_ and an objective lens with an angular magnification of f_1_/f_2_, in which f1 is the focal length of L_3_ and f_2_ is the focal length of the objective lens. After compression, the half conical angle of the compressed Bessel beam, *θ*, increases to 38.15° when f_1_/f_2_ = 100. The diameter of the Bessel beam core is theoretically expressed as 2.405λ/(πsin*θ*) from bottom to peak and 1.125λ/(πsin*θ*) in full width half maximum (FWHM), which can be calculated as 638.28 nm and 298.57 nm, respectively. The silica sample is put on a piezo moving stage in X-Y-Z directions with 10 nm resolution. The longitudinal and radial profiles of the Bessel beam after compression are shown in Figure 1b,c, respectively, which are acquired by collecting the reflecting light. It can be seen from Figure 1b that most of the beam energy concentrates in the axis of the Bessel beam, namely the center spot. Along the propagation direction, the Bessel beam maintains the same shape with a distance of ~15 μm. The intensity distribution inside the center spot along the propagation direction is shown in Figure 1c. The longitudinal intensity maintains over half the value of the peak from the position of 2.30 μm to 11.77 μm. The full width for FWHM is 9.47 μm. The radial profile of the Bessel beam is shown in Figure 1d, in which the ring structures around the center spot of the Bessel beam are evident. The diameter of the center spot of the Bessel beam is 680 nm from bottom to peak and 391 nm for FWHM.

## 3. Results

**Figure 2:**
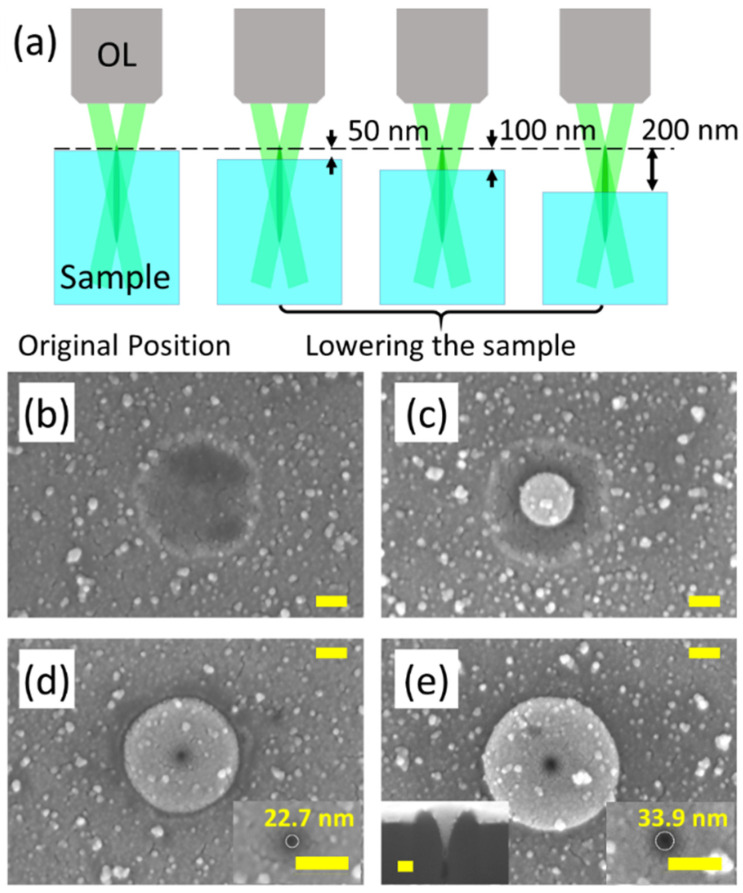
(**a**) the scheme of changing the relative position between the Bessel beam and the sample surface; (**b**–**e**) scanning electron microscopy (SEM) image of surface morphology (**b**) at the “original position” and when lowering the sample on the moving stage by (**c**) 50 nm, (**d**) 100 nm, and (**e**) 200 nm along the beam propagation direction; the pulse energy is 0.8 μJ. The scale bar: 100 nm.

Figure 2 shows the surface morphology of silica ablated by the tightly focused Bessel beam with the sample positions changing. The scheme of the experiments is shown in Figure 2a. When the Bessel beam is deeply buried inside the silica sample, only a shallow crater exists on the surface, as shown in Figure 2b. The diameter of the crater is 384 nm on average. The position corresponding to Figure 2b is defined as the “original position” and then we gradually lower the piezo stage with a smallest step length of 10 nm. The Bessel beam is “elevated” from the sample relatively. When lowering the piezo stage only by 50 nm, a nanoplate begins to appear in the middle of the crater, as shown in Figure 2c. The average diameter of the nanoplate is 173.07 nm and the average diameter of the crater is 393.71 nm. Compared with that in Figure 2b, the size of the crater does not change too much. More evident modification of the surface morphology appears when lowing the piezo stage by 100 nm from the “original position”. As shown in Figure 2d, a plate-hole structure can be observed. The diameter of the plate is 372 nm and the diameter of the center hole is 22.7 nm. The incident wavelength of the fs laser is 515 nm so the diameter of the nanohole could almost achieve ~1/25 *λ*. The edge of the crater that exists in Figure 2b,c can be still observed. Similar plate-hole structures can also be discovered in Figure 2e, corresponding to lowering the piezo stage to 200 nm from the original position. However, the sizes of the nano-hole and plate are much larger than that in Figure 2d. The nanohole in the center has a diameter of 33.9 nm and the surrounding plate increases to 576.1 nm in diameter. In addition, the crater structure is totally covered by the plate structures in Figure 2e. A cross-section image of the plate-hole structures in Figure 2d,e is shown in the inserted image of Figure 2e. The “plate” is a funnel shape structure from a cross section view with a nano-hole in the bottom of the funnel.

The generation of the surface morphologies in Figure 2 can be explained by the numerical results of energy deposition based on the model in Ref. [25,26]. The interaction between the incident Bessel beam and the silica material consists of the unidirectional envelope propagation, the ionization of silica, and the energy deposition induced by the free electrons. The unidirectional envelope propagation is described as:

(1) $$\frac{\partial \tilde{E}}{\partial z}=\frac{i}{2k}\left(\frac{\partial^{2}}{\partial r^{2}}+\frac{1}{r}\frac{\partial }{\partial r}\right)\tilde{E}-i\frac{k^{\prime \prime }}{2}\frac{\partial^{2}\tilde{E}}{\partial \tau^{2}}+ik_{0}n_{2}{\left|\tilde{E}\right|}^{2}\tilde{E}-\frac{\sigma }{2}\left(1+i\omega_{0}\tau_{c}\right)\rho \left(\tau \right)\tilde{E}-\frac{1}{2}\frac{W_{PI}\left(\left|\tilde{E}\right|\right)U_{i}}{{\left|\tilde{E}\right|}^{2}}\tilde{E}$$

In Equation (1), $\tilde{E}$ is the electric field. $k^{\prime \prime }\equiv \partial^{2}k/\partial \omega^{2}$ donates the group velocity dispersion. $k_{0}n_{2}{\left|\tilde{E}\right|}^{2}$ donates the second-order nonlinear effects, such as self-focusing. $\left(\sigma /2\right)\left(1+i\omega_{0}\tau_{c}\right)\rho \left(\tau \right)$ donates free electrons’ contribution to the light field and $\left(1/2\right)W_{PI}\left(\left|\tilde{E}\right|\right)U_{i}/{\left|\tilde{E}\right|}^{2}$ donates multi-photon absorption. The temporal evolution of the free electrons is described through the temporal free electron density ρ(τ) and the laser intensity I, shown as:

(2) $$\frac{\partial \rho \left(\tau \right)}{\partial t}=\left(W_{PI}\left(\left|\tilde{E}\right|\right)+\frac{\sigma I}{U_{i}}\rho \left(\tau \right)\right)\left(1-\frac{\rho \left(\tau \right)}{\rho_{max}}\right)-\frac{\rho \left(\tau \right)}{\tau_{r}}$$

In Equation (2), $W_{PI}\left(\left|\tilde{E}\right|\right)$ describes the contribution of multiphoton ionization and $\sigma I\;\rho \left(\tau \right)/U_{i}\;$ describes the contribution of avalanche ionization. $\rho \left(\tau \right)/\tau_{r}$ describes the lifetime of the free electrons.

**Figure 3:**
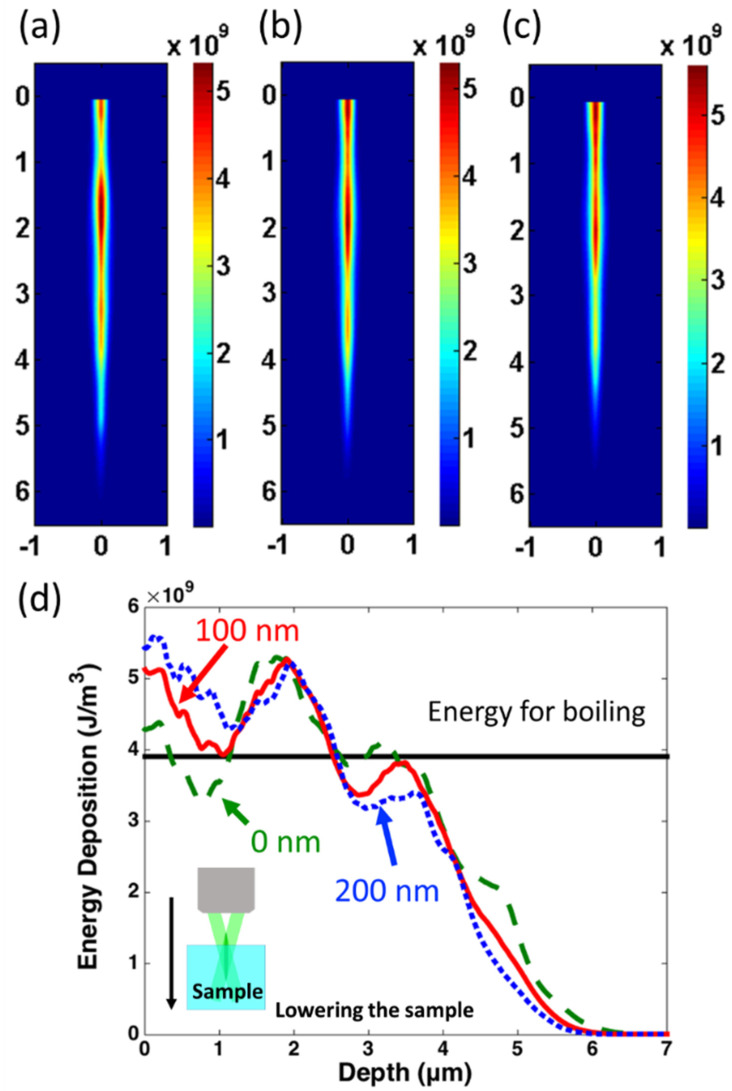
Simulation results for energy deposition by lowering the sample of (**a**) 0 nm, (**b**) 100 nm and (**c**) 200 nm along the beam propagation direction; tick label unit in (**a**–**c**): μm, tick label unit for color bar: J/m^3^. The 0 nm position corresponds to the surface of the silica sample, (**d**) Energy deposition along the Bessel beam axis inside silica with different beam locations; green and dashed line: 0 nm; red and solid line: 100 nm; blue and dotted line: 200 nm; black and horizontal line: energy density for boiling.

The energy depositions depending on the sample positions are shown in Figure 3a–c. The intense incident laser induces strong nonlinear absorption inside the silica. The electrons are ionized rapidly due to multi-photon ionization and avalanche ionization. The following incident light is strongly absorbed by these free electrons during the light propagation inside the silica. As a result, the energy absorption efficiency deep inside the silica will also drop, which finally leads to the drop in the energy deposition density. For the area in the vicinity of the surface; however, the laser beam incident occurs on the surface and then is deposited near the surface directly, without propagating inside the silica. Therefore, the energy deposition efficiency on the surface is relatively higher than that inside the material. Finally, the energy deposition density shows evident dips beneath the silica surface, generating two relatively high energy density spots. One is on the surface and the other is deeply buried inside the silica. The dip in energy deposition density is especially obvious in Figure 3a. Here, the Bessel beam position in Figure 3a in the numerical simulation corresponds to the “original position” in Figure 2. The axial energy depositions on the center spot of the Bessel beams are shown in Figure 3d. For the Bessel beam at the “0 nm position”, the hot spot on the surface and inside the material both exceed the energy for boiling. However, in the area between the two hot spots, the energy density is significantly below it. For the hot spot on the surface, the temperature exceeds boiling and the superficial material will evaporate within hundreds of ps. The removed material generates a shallow crater such as that in Figure 2a. For the interior hot spot, however, the high-temperature area is surrounded by relatively cool and dense materials. In this condition, the hydrodynamic activities such as expansion are confined beneath, and the superficial morphologies are not affected. By lowering the sample by 100 and 200 nm, higher energy deposition occurs on the surface with the elevation of the Bessel beam. Compared with that in “original position”, the temperature at the deposition dip between the two hot spots increases, as is shown from the solid and dotted curves in Figure 3d. In this condition, the interior material maintains a higher temperature compared with that of the “original position”, which leads to expansion and ejection out of the surface through the window provided by the surface hot spot. Thermal expansion can explain both the top-view and the cross-section images in Figure 2d,e, in which smooth profiles of the “plate-hole” or “funnel-hole” structures can be observed and no sharp edges appear. The interior material ejection also occurs when the temperature between the two hot spots increases over boiling, which can explain the nano-hole structures in the center of the nano-plates. In addition, surface evaporation is a process much faster than both the expansion and ejection, which can explain the coexistence of the crater structures, and the “plate” or the “plate-hole” structures in Figure 2c–e.

## 4. Discussion

By adjusting the interior and surface interaction during the femtosecond Bessel beam ablation on silica, a series of nanostructures can be acquired. By modifying the Bessel beam position within a range of 200 nm along the Bessel beam propagating direction, crater, nanoplate-crater, and nanoplate-nanohole structures appear consecutively, which is very sensitive to the relative position of the Bessel beam and the silica surface. Among these special nanostructures acquired on the silica surfaces, the nanoplates can be potentially applied in the optical trapping of nanoparticles [27]. The nanohole structures with ~20 nm diameter can be applied in the rapid detection of nuclear acids, viruses, and other biomolecules [28,29]. The generation of the nanoplate-nanoholes and other structures observed are the interaction results between surface and interior energy deposition, which can be concluded as a surface window assisted nano-drilling (SWAN) mechanism. The higher energy deposition on the “surface” leads to rapid material removal, providing the space for interior material expansion. Then, “assisted” by the “window” opened by the surface hot spot, the interior materials heated by inside the hot spot can expand to the surface and be ejected. As a result, the co-action of surface material removal and interior material jet finally leads to the “nano-drilling” structures shown in this paper. Theoretical analysis is confined here to energy deposition processes. Newly developed ultrafast imaging and super-resolution microscopy techniques may also help in understanding the mechanism of different surface morphology [30,31,32]. This work holds physical significance in investigating laser-material interaction and wide potential applications.

## Data Availability

Not applicable.
